# Fetometry in Arabian horses

**DOI:** 10.3389/fvets.2025.1689769

**Published:** 2025-10-23

**Authors:** Ahmed Ali, Derar R. Derar, Ahmad A. Alaeyeari, Yousef M. Alharbi

**Affiliations:** ^1^Department of Clinical Sciences, College of Veterinary Medicine, Qassim University, Buraydah, Saudi Arabia; ^2^Department of Medical Biosciences, College of Veterinary Medicine, Qassim University, Buraydah, Saudi Arabia

**Keywords:** horse, Arabian, pregnancy, gestational age, regression model, fetal sex, ultrasound

## Abstract

**Introduction:**

This study aimed to monitor intrauterine fetal growth, establish predictive equations for gestational age estimation, and determine the optimal period for fetal sexing in Arabian horses using ultrasonography.

**Materials and methods:**

Seven Arabian mares were monitored from insemination to parturition using transrectal ultrasonography. The following fetal and embryonic parameters were measured: embryonic vesicle (EV), crown–rump length (CRL), biparietal diameter (BIP), stomach diameter (STD), chest depth (CHD), abdominal diameter (ABD), kidney length (KDL), eyeball diameter (EBD), and eye lens length (ELL). Key developmental milestones were recorded, including detection of the EV, embryo proper, organization, and ossification.

**Results:**

The EV, embryo proper, organization, and ossification were visualized at days 10.3 ± 0.5, 21.3 ± 1.3, 33.6 ± 2.0, and 57.7 ± 3.5, respectively. All biometric parameters, except CRL, ELL, and KDL, exhibited strong linear correlations with gestational age (P < 0.0001). Fetal sexing was feasible between days 56 and 161, with maximum accuracy (98%) and applicability (64.9%) between days 105 and 133.

**Discussion and conclusion:**

Ultrasonographic monitoring provides reliable parameters for assessing fetal growth and estimating gestational age in Arabian horses. The period between days 105 and 133 of gestation represents the optimal window for accurate fetal sex determination. These findings enhance reproductive monitoring and breeding management practices in Arabian horse herds.

## 1 Introduction

Estimation of gestational age is essential for the maintenance of high levels of reproductive efficiency. Such information would allow producers to predict parturition dates, group animals based on their nutritional needs, and identify fetuses at an increased risk of perinatal disease (1, 2). Moreover, due to the wide range of gestational lengths (320–360 days) and the lack of clear signs of impending parturition, predicting a mare’s foaling date is critical in stud farm management (3, 4).

Intrauterine fetal growth is a complex biological process influenced by environmental factors, breed, maternal size, age, and parity (5–7). Arabian horses are typically small and refined. According to the United States Equestrian Federation’s breed standard (8), Arabians stand between 145 and 155 cm tall. Despite the traditional cutoff height of 147 cm between a horse and a pony, all Arabians, regardless of height, are classified as “horses.”

Using sonography, it has become possible to study the development and growth of the fetal organs and parts without endangering pregnancy in different animal species (9–12). Moreover, the development of regression models for the prediction of gestational age and time of parturition has been reported for different breeds of horses, including Dutch Warmblood (13), Thoroughbred (14), Standardbred (15), and Quarter breed (2, 16). Because these breeds differ in size from Arabian horses, these models may be ineffective for predicting gestation and foaling dates in Arabian horses.

Identifying the gender of a fetus can help manage horse reproduction. There are two ultrasound techniques available. The first was used for early diagnosis, which uses the transrectal approach and is dependent on the location of the genital tubercle (GT) (17). The second was performed during mid-gestation and used a combination of transrectal and transabdominal ultrasonographic approaches, depending on the observation of the penis/prepuce, gonads, mammary gland, and genital tubercle location (18).

This study aimed to develop ultrasonographic regression models for gestational age estimation and to evaluate the feasibility and accuracy of fetal sex determination in Arabian horses.

## 2 Materials and methods

All examinations were carried out without sedation and were well tolerated by the mares. Gentle handling reduced stress and resulted in no adverse effects or complications. The study met institutional ethical standards and was approved in accordance with the Deanship of Research at Qassim University guidelines.

### 2.1 Animals and management

A group of 12 maiden Arabian mares aged 42–48 months, weighing between 395 and 460 kg, were synchronized for estrus in March 2024. Estrous synchronization was performed using daily oral progesterone (Regumate, altrenogest 0.22%, 0.044 mg/kg, Intervet Inc., Merck Animal Health, NJ, USA) for 10 days, with prostaglandin F2α (PG; 250 μg cloprostenol; EstrumateTM, Essex Tierarznei, Germany) administered on the 10th day. The mares ovulated 6 days (*n* = 9), 8 days (*n* = 2), and 13 days (*n* = 1) after PG injection. The mares were artificially inseminated with frozen semen from one stallion immediately after ovulation. Only seven mares were confirmed pregnant and included in the study. These mares were clinically healthy, aged 36 ± 3.9 months, weighing 428.57 ± 25 kg, and had body condition scores of 6.87 ± 0.69 using a scale from 1 (poor) to 9 (extremely fat) (19). The day of insemination was considered day 0 of pregnancy. The mares were stabled in closed pens at one stud farm in the Qassim region (central Saudi Arabia; longitudes 43°42′E to 43°90′E and latitudes 26°10′N to 26°45′N). The mares were provided with a balanced diet and had *ad libitum* access to water.

### 2.2 Ultrasonographic examination

Sequential transrectal ultrasonographic examinations were performed on the seven pregnant mares, daily from days 0 to 35, once weekly between days 35 and 91, and every 2 weeks from day 91 until parturition. The examinations were performed while the mares were in a standing position secured in a stanchion. Real-time, B-mode diagnostic ultrasound equipment (Sonoscape X3V, Hamburg, Germany) attached with multiple frequency linear transducers was used for scanning. The rectum was evacuated of feces, and the mare’s uterus was examined by palpation under the rectum. The ultrasound transducer was then inserted into the rectum, and the uterine contents were examined. The transducer was swept from left to right across the uterus, starting at the internal cervical os and gradually proceeding cranially. The required images were frozen, and maximum measurements were obtained.

### 2.3 Earliest detection of the amniotic vesicle, embryo proper, organization, and ossification

The earliest time of detection of the AV and embryo proper was recorded. The time of embryo organization into the head, body, and limbs was detected. Increased echogenicity in the area of suspected fetal bone formation was considered the onset of ossification (20, 21). Turbidity of the fetal fluids was observed. Fetal presentation (anterior vs. posterior) was recorded.

### 2.4 Ultrasonic fetometry

During each ultrasonographic examination (duration 10–15 min per mare), attempts were made to measure the following fetometric parameters: amniotic vesicle diameter (AVD, the maximum diameter of the amniotic vesicle); crown-rump length (CRL, a straight line between the fetal crown and the origin of the tail); biparietal diameter (BPD, the widest distance between the outer borders of the cranium); chest depth (CHD, the dorso-ventral distance just caudal to the apex of the heart, representing depth of thorax); abdominal diameter (ABD, the maximum diameter of the abdomen at the insertion of the umbilical cord); stomach diameter (STD, the largest intraluminal diameter of the stomach); eyeball diameter (EBD, the longest dimension of the vitreous body from the medial sclera to lateral sclera); length of the eye lens (ELL, the maximum length of the eye lens); and kidney length (KDL, the largest kidney length from anterior to posterior poles). Required images were frozen on the monitor of the ultrasound scanner, and fetal dimensions were measured at their maximum using an electronic caliper.

### 2.5 Prenatal fetal sex assessment

On each ultrasonographic occasion, trials were made to view the fetus in the frontal plane by holding the transducer in a position in which the fetal head, forelimbs, umbilical cord, hind limbs, and tail appeared as spots in one view. The area from the umbilical cord to the tail was scrutinized to identify the appearance of GT, the mammary gland, scrotum, or phallus. The fetus was identified as male when the GT was found immediately cranial to the hind limb or caudal to the umbilical cord’s abdominal attachment or when the phallus was observed. When the GT was observed behind the hind limbs or near the base of the tail, or when the mammary gland was visible, the fetus was deemed to be female (17, 18). The expected prenatal fetal sex was compared with that observed at birth.

### 2.6 Statistical analysis

Stepwise regression and correlation models were fitted to evaluate the relationship between gestational age and each of the studied parameters. The various measurements were considered to be dependent on gestational age. A 5% significance level was used. For data analysis, version 18.0 of the IBM SPSS computer program was used. Data were expressed as mean ± SD or percentages.

## 3 Results

### 3.1 Pregnancy duration and the weights and heights of mares, foals, and the placenta

The seven mares underwent 543 ultrasonographic examinations, with an average of 77.57 ± 1.9 examinations per mare (range 76–81). There were no adverse effects from repeated ultrasound examinations. All mares delivered normally at term. The average pregnancy duration was 350.43 ± 13.96 days (range: 339–370 days). Table 1 shows the average dam weight and fetal wither height at birth. Positive relationships were detected between pregnancy duration and foal weight (*r* = 0.96, *p* = 0.001), dam weight and foal weight (*r* = 0.98, *p* = 0.0001), and dam height and foal height (*r* = 0.99, *p* = 0.0001). The average placental weight was 3.19 ± 0.36 kg (range: 2.9–3.7 kg).

**Table 1:** Dam and foal weight and wither height (mean ± SD) at birth.

Item	Dam	Foal	Foal/Dam ratio
Weight (kg)	383.82 ± 54.98 (range 304.9–425)	36.01 ± 14.34 (range 27.5–57)	9.45 ± 3.46% (range 9.12–14.21%)
Wither height (cm)	158.57 ± 2.45 (range 155–160)	103.43 ± 11.22 (range 87–110)	65.14 ± 6.16 (range 56.13–68.75)

### 3.2 First detection of embryonic vesicle, embryo proper, and fetal organization and ossification

**Figure 1:**
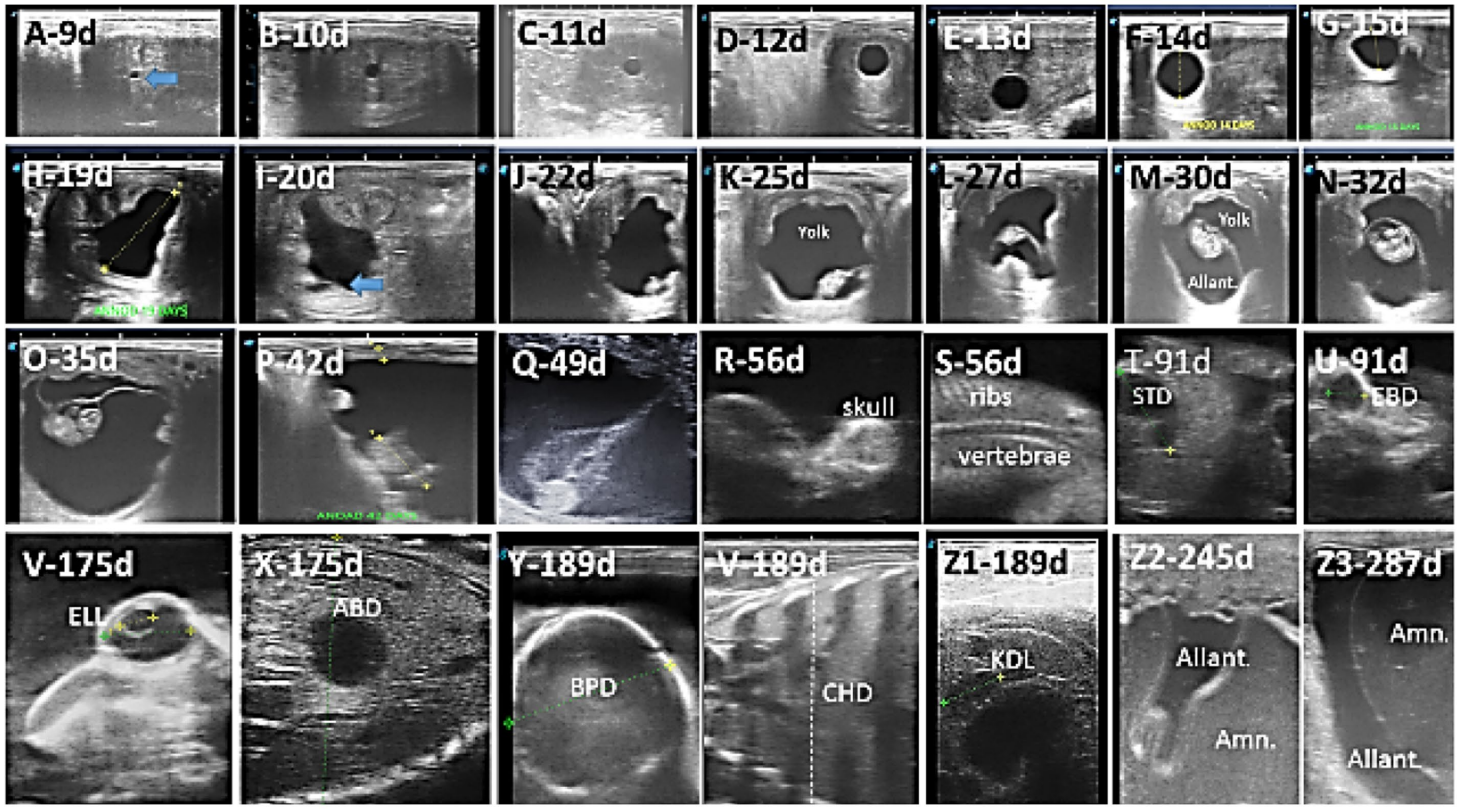
Ultrasound images depict the different developmental stages in the Arabian horse conceptus. **(A–H)** Beginning of detection and change in the size and shape of the embryonic vesicle, EV; **(I–Q)** conceptus is first observed, with changes in length and position within the embryonic vesicle, as well as changes in yolk and allantotic fluid volume; **(M–O)** development of the head, body, and limbs; **(R,S)** start of ossification in skull, vertebrae, and ribs; **(T–Z1)** measure the stomach diameter (STD), eyeball diameter (EBD), lens (ELL), abdominal diameter (ABD), biparietal diameter (BPD), chest depth (CHD), and kidney length (KDL); **(Z2,Z3)** increased amniotic fluid and allantoic.

The EV was first observed as circumscribed hyperechogenic in the central part of the uterine horn on day 10.29 ± 0.49 (range, days 10–11) after insemination. The EV remained exactly round until day 17.43 ± 0.53 (range, days 17–18) of gestation but became irregular in shape (Figures 1A–H).

The embryo proper was first detected as a tiny hyperechogenic spot at the base of the AV on day 21.29 ± 1.25 (range, days 20–23) of gestation. The embryo moved gradually upward to reach the middle of the EV on day 31.14 ± 2.12 (range, days 28–33) of gestation. It continues to climb upward to reach the upper third of the AV on day 34.86 ± 0.38 (range, days 34–35). Then, it moves down, attached to the umbilical cord, to reach the floor on day 56.73 ± 3.78 (range, days 55–60) of gestation (Figures 1I–Q).

Differentiation of the embryo into head, body, limbs, and tail was noted on day 33.57 ± 1.99 (range, days 30–35) of gestation (Figures 1M–O).

Beginning of ossification was observed in the head, ribs, and vertebrae on day 57.71 ± 3.45 (range 55–62 days) of gestation (Figures 1R,S).

The amniotic fluid contained no reflecting particles and gave an almost black image until day 133.42 ± 11.2 (range, days 120–143), when many echogenic spots were observed whirling around in it. Definite turbidity was observed at day 178.29 ± 3.86 (range, days 172–185) (Figures 1Z2,Z3). The allantoic fluid remained clear without any echogenic particles until day 323 ± 9.995 (range, days 305–332) of gestation, when a few echogenic flakes appeared to move within it.

### 3.3 Ultrasonographic fetometry

**Figure 2:**
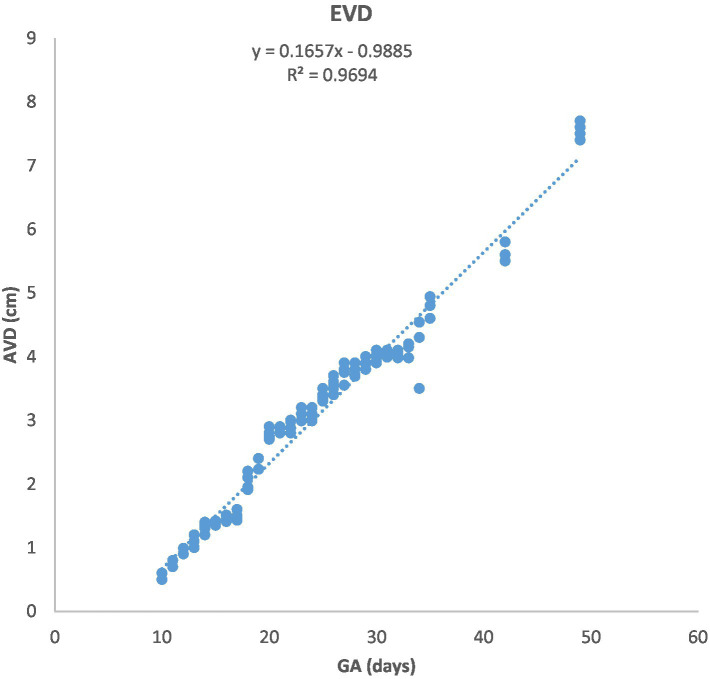
Growth of the amniotic vesicle diameter (EVD) in Arabian mares (*n* = 7). EVD increased linearly with gestational age (GA). Regression analysis showed a highly significant correlation (*p* < 0.0001, *R*^2^ = 0.9694).

**Figure 3:**
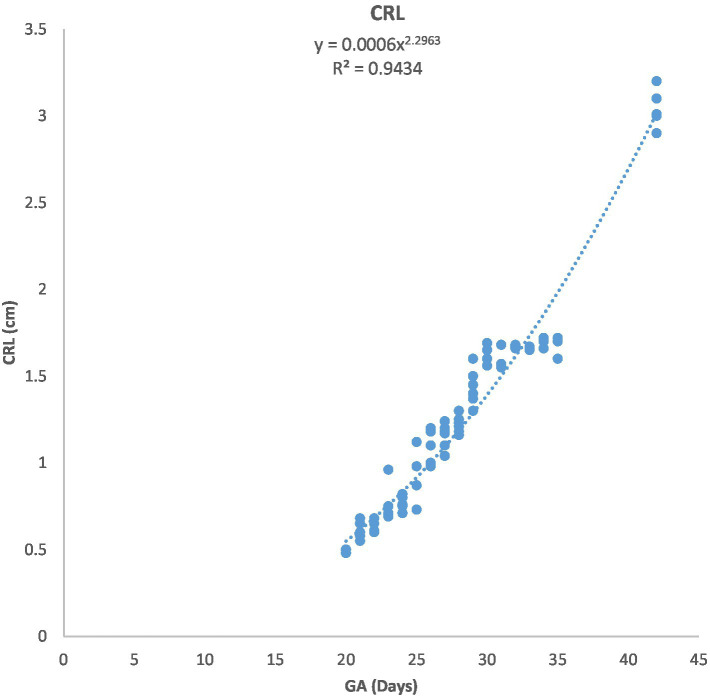
Changes in crown-rump length (CRL) during pregnancy in Arabian horses (*n* = 7). CRL growth was initially slow, then accelerated with advancing GA. Correlation was highly significant (*p* < 0.0001, *R*^2^ = 0.9434).

**Figure 4:**
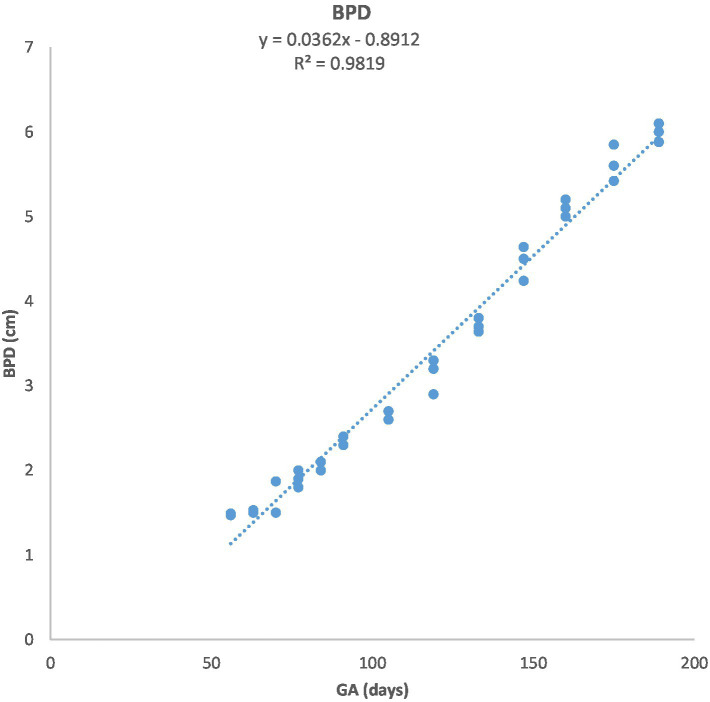
Biparietal diameter (BPD) growth in Arabian horses (*n* = 7). BPD increased linearly with GA. Regression showed a highly significant correlation (*p* < 0.0001, *R*^2^ = 0.9824).

**Figure 5:**
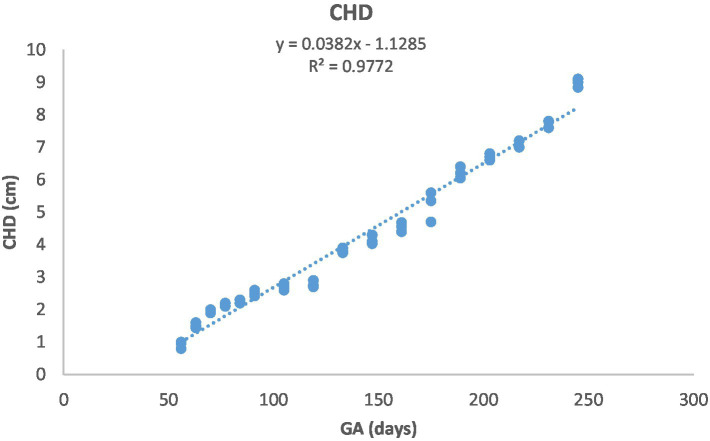
Chest depth (CHD) development during pregnancy in Arabian horses (*n* = 7). CHD grew linearly with GA, showing a highly significant correlation (*p* < 0.0001, *R*^2^ = 0.9772).

**Figure 6:**
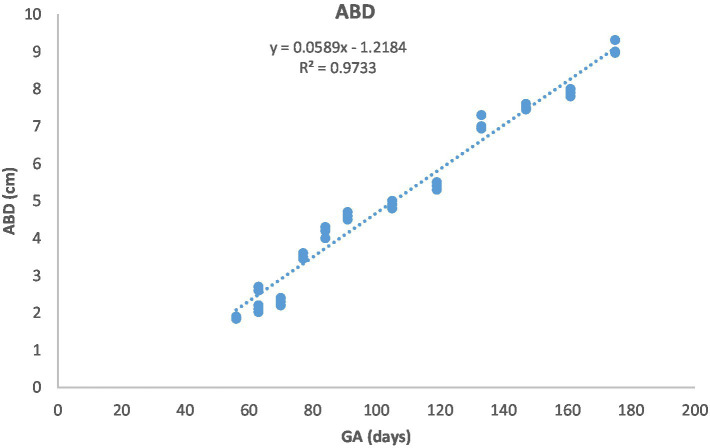
Abdominal diameter (ABD) growth in Arabian horses (*n* = 7). ABD increased linearly with GA. Regression was highly significant (*p* < 0.0001, *R*^2^ = 0.9733).

**Figure 7:**
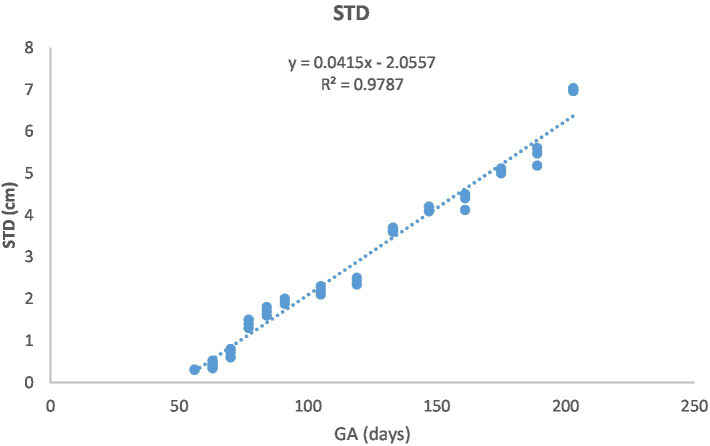
Stomach diameter (STD) changes during pregnancy in Arabian horses (*n* = 7). STD grew linearly with GA, with a highly significant correlation (*p* < 0.0001, *R*^2^ = 0.9787).

**Figure 8:**
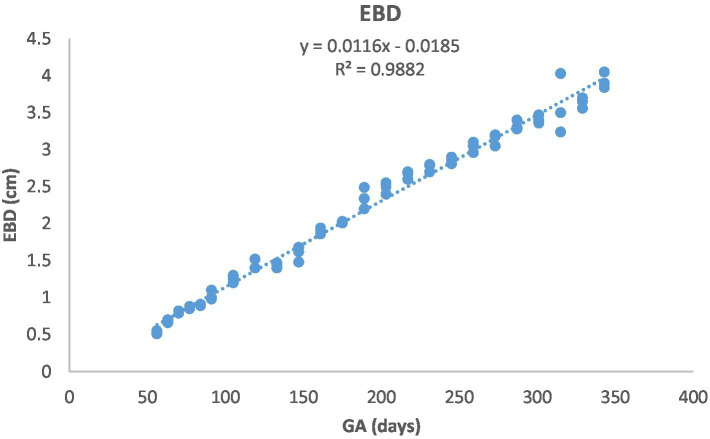
Eyeball diameter (EBD) development in Arabian horses (*n* = 7). EBD increased linearly with GA. Correlation was highly significant (*p* < 0.0001, *R*^2^ = 0.9882).

**Figure 9:**
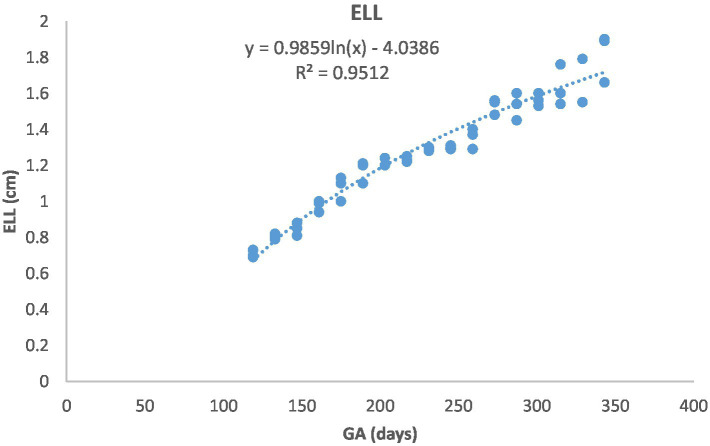
Eye lens length (ELL) growth in Arabian horses (*n* = 7). ELL followed a curvilinear pattern: rapid growth early, slowing later. Correlation was highly significant (*p* < 0.0001, *R*^2^ = 0.9512).

**Figure 10:**
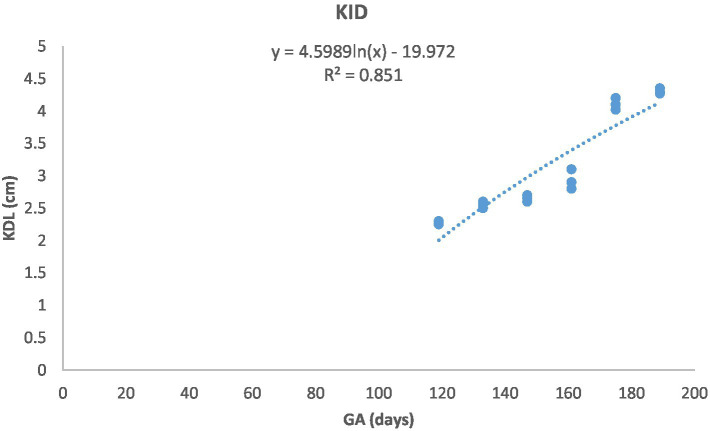
Kidney length (KDL) changes during pregnancy in Arabian horses (*n* = 7). KDL showed a curvilinear pattern, with rapid early growth followed by a slower increase. Correlation was highly significant (*p* < 0.0001, *R*^2^ = 0.851).

Figures 1T–Z1 depicts the ultrasonographic estimation of the length, diameter, and depth of various fetal organs and parts. They grew at different rates during the observation periods. Some grew steadily (EVD, BPD, CHD, ABD, STD, and EBD), some started slowly and then increased rapidly as the pregnancy progressed (CRL), and some started at high rates and then decreased in growth as the pregnancy progressed (ELL and KDL). Charts of the growth patterns of these organs/parts are illustrated (Figures 2–10). The regression and correlation analyses between gestational age and the studied parameters were highly significant (*p* < 0.0001).

Accessibility of the different fetal organs and parts for ultrasonic examinations depended on the day of gestation and the technique of examination. The AVD and CRL were accessible only during early pregnancy; subsequently, they became excessively lengthy to be displayed effectively on the screen in their entirety. The BPD, CHD, ABD, STD, EBD, and ELL could be scanned during mid-gestation. EBD and ELL were within the range of the ultrasound beam during mid-gestation and late-gestation. Table 2 shows the accessibility of different fetal parts and organs during the total gestational period.

**Table 2:** Accessibility of different embryo/fetal parts and organs using trans-rectal ultrasonography in pregnant Arabian mares (*n* = 7).

Embryo/fetal parameters	Days of observations	Number of observations	Accessibility rate (The possibility of viewing it with ultrasound)
AVD	10–49	196	56%
CRL	20–42	126	36%
BPD	56–189	91	26%
CHD	56–245	119	34%
ABD	56–175	84	24%
STD	56–203	98	28%
KDL	11–189	42	12%
EBD	56–343	175	50%
EEL	119–243	119	34%

### 3.4 Fetal presentation

**Figure 11:**
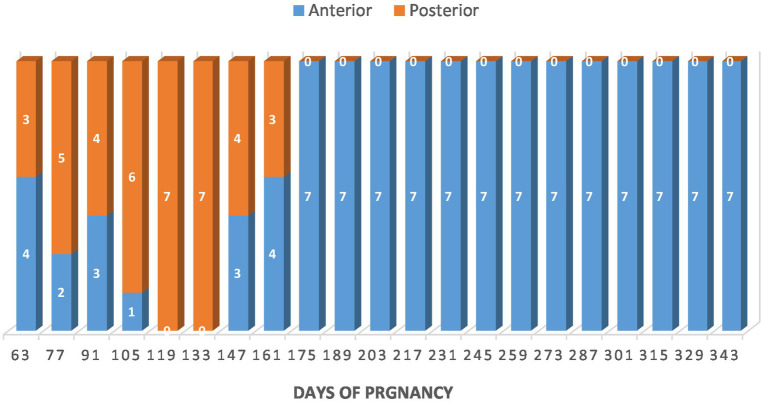
Fetal presentation in Arabian mares (*n* = 7). Fetuses changed position frequently until day 161 of gestation; from day 175, all were in anterior presentation. Two weeks before parturition, all were dorso-pubic with the forelimbs flexed at the carpal joints.

The fetuses changed their presentation frequently until day 161 of pregnancy. From day 175 onward, all fetuses were in the anterior presentation (Figure 11). Two weeks before parturition, all fetuses were in a dorso-pubic position, with the two forelimbs flexed at the carpal joints.

### 3.5 Fetal sexing

**Figure 12:**
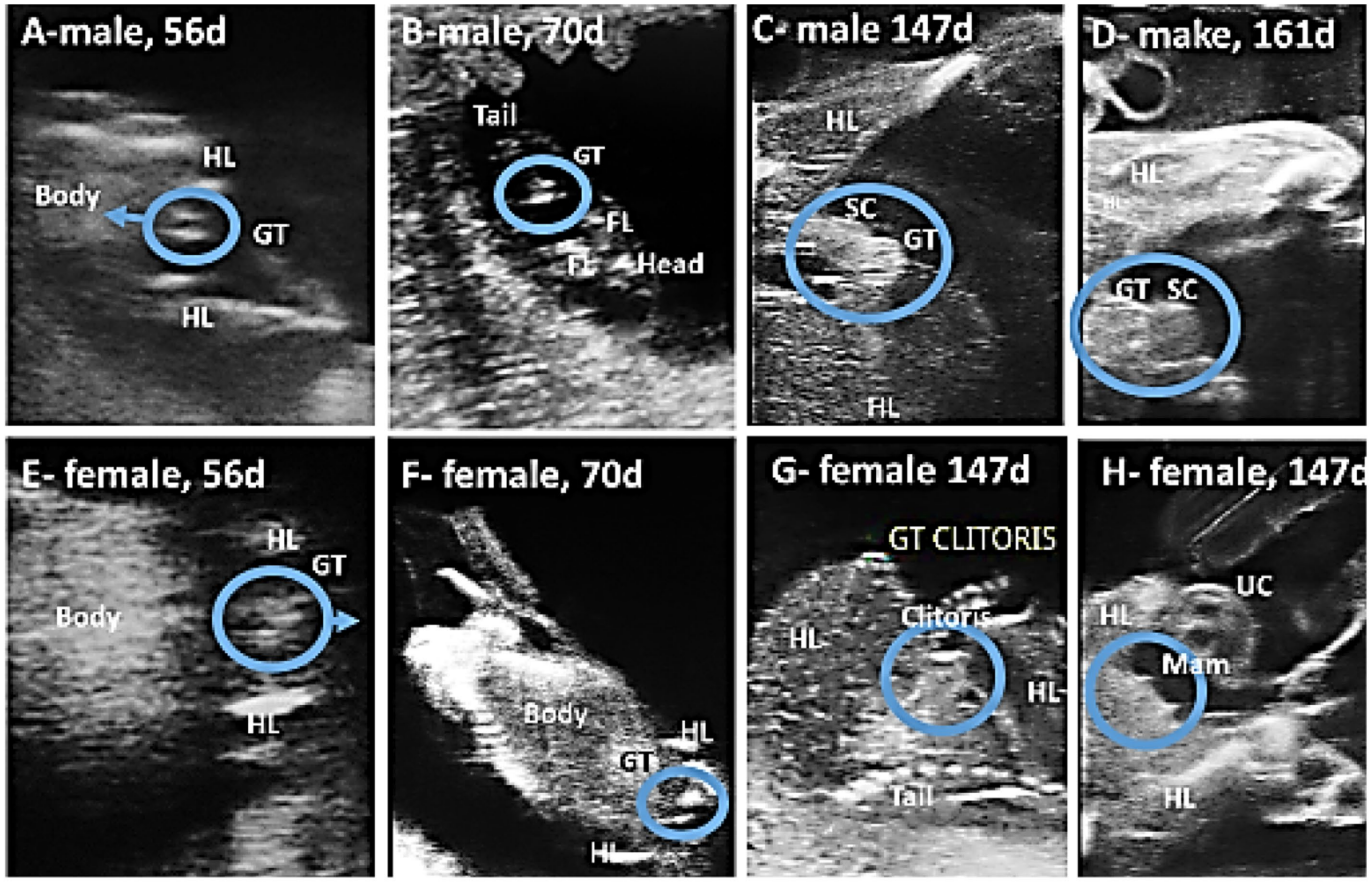
Using ultrasound to predict the gender of camel fetuses. In the ventral view of the male fetus **(A,B)**, the genital tubercle (GT) advances anteriorly until it reaches the junction of the umbilical cord (UC) and the fetus, whereas in the female fetus **(E,F)**, the genital tubercle is located posterior to the hind limbs (HL) and gradually moves backward toward the tail. As pregnancy progresses, the scrotum (SC) appears between the hind limbs in males **(C,D)** and opposite the clitoris and mammary glands (Mam) in females **(G,H)**.

The fetus’s sex can be determined between the 56th and 161st days of gestation, with the best timeframe falling between days 105 and 133 (Table 3). Fetal sexing was determined by observing the GT in early pregnancy and the scrotum or mammary glands in mid-gestation, while the fetus was in frontal view. In early pregnancy (56–92 days), male fetuses (*n* = 4) had a hyperechogenic, flattened, oval, or round genital tubercle (phallus) located cranial to the hind limbs and at the abdominal attachment of the umbilical cord (Figures 12A,B). The genital tubercle (clitoris) was found behind the hind limbs and near the base of the tail in three female fetuses (Figures 12E,F). Fetal scrotum or the mammary glands may be visible between the two hind limbs by day 141 ± 7.48 of gestation (Figures 12C,D,G,H). Fetal sexing was applicable on 50 of 77 occasions (64.94%). The gender of the fetus was correctly predicted on 49 of 50 occasions (overall accuracy 98%).

**Table 3:** Prediction of fetal gender using transrectal ultrasonography in Arabian horse (*n* = 7).

Day of pregnancy	Mares							Applicability (%)	Accuracy (%)
	M1	M2	M3	M4	M5	M6	M7		
56	Male	Male	Female	Male	n/a	Female	n/a	5/7 (71.43)	5/5 (100)
63	Male	Male	Female	Male	n/a	Female	n/a	5/7 (71.43)	5/5 (100)
70	n/a	n/a	Female	Male	fem	n/a	n/a	3/7 (42.85)	3/3 (100)
77	n/a	n/a	n/a	Male	fem	n/a	n/a	2/7 (28.57)	2/2 (100)
84	n/a	Male	n/a	n/a	n/a	n/a	Male	2/7 (28.57)	2/2 (100)
91	Male	n/a	Female	Male	n/a	Female	n/a	4/7 (57.14)	4/4 (100)
105	Male	Male	Female	Male	fem	Female	Male	7/7 (100)	7/7 (100)
119	Male	Male	Female	Male	Male	Female	Male	7/7 (100)	6/7 (85.71)
133	Male	Male	Female	Male	fem	Female	Male	7/7 (100)	7/7 (100)
147	Male	Male	Female	Male	n/a	Female	n/a	5/7 (71.43)	5/5 (100)
161	n/a	Male	Female	n/a	n/a	Female	n/a	3/7 (42.86)	3/3 (100)
Fetal sex at birth	Male	Male	Female	Male	Female	Female	Male		
Total	8/11 (72.72)	8/11 (72.72)	9/11 (81.81)	7/11 (63.63)	6/11 (54.54)	8/11 (72.72)	5/11 (45.45)	50/77 (64.94)	49/50 (98)

## 4 Discussion

The study’s underlying assumption was that Arabian horses would exhibit smaller fetal measurements and potentially distinct regression model characteristics compared to those of other horse breeds. To confirm this, the birth weight of Arabian foals in this study was lower than that of other breeds (5, 22–24), and even lower than that of Arabian horses in other countries (25), perhaps because the mares in the study were giving birth for the first time. In fact, intrauterine fetal growth and subsequent birth weight constitute a complex biological phenomenon, influenced by the placenta and impacted by environmental factors, genetic and epigenetic mechanisms, and maternal characteristics, including size, age at conception, and parity (7, 26, 27).

Although the timing of detection of the EV, embryo proper, turbidity of the fetal fluid, loss of the EV’s round shape, mobility of the conceptus within the EV, and change in fetal presentation were similar to those previously described in other breeds of mares (13–15, 28), breed-specific differences in ultrasonic biometric thresholds were observed. In Thoroughbred horses, the CRL increased linearly from weeks 5 to 13 (14), whereas in this study, it exhibited a curvilinear growth pattern with increasing growth rate as pregnancy progressed. The EBD in this study developed linearly from days 50 to 350, whereas in Thoroughbreds, it increased curvilinearly with decreasing growth rate as pregnancy progressed (14). Furthermore, KDL developed linearly in Thoroughbreds (14, 29), whereas it exhibited a curvilinear growth pattern in Arabian mares, with decreasing growth rate as gestation progressed. The values of these parameters were consistently lower in Arabian horses than in Thoroughbred horses.

The EBD was the most reliable predictor of fetal age in Arabian horses throughout gestation. In Quarter Horses, BPD, eye volume, and femur length were the best predictors of fetal age between 100 and 200 days of gestation, BPD and eye volume between 200 and 300 days, and eye volume after 300 days (29). Consistent with our findings, the orbital diameter emerged as the most reliable metric for assessing embryonic and fetal physiological development, with a strong correlation with gestational age in small pony (30) and donkey (31). In general, the availability of the various fetal parts for scanning depended on the method of examination, the type of transducer, and the fetal position (13, 14, 16).

In this study, the timing of embryo organization, ossification, and detection of various fetal parts and organs differed in some aspects from what was observed grossly. For example, the limbs of equine embryos were visible grossly at 35–40 days, the optical vesicle on day 25, the kidney on days 36–38, the stomach on day 25, and the ribs on day 25 of gestation (32, 33). Ultrasonography revealed ossification in buffalo embryos between days 56 and 70 (20) and in camel embryos between days 49 and 63 (21), which aligns with observations in Arabian horses. The timing of organization and ossification may help determine fetal age.

As gestation progresses, most equine fetuses assume an anterior presentation, likely because the fetal body becomes longer than the width of the amniotic cavity. Bucca et al. (34) reported that posterior and transverse presentations occur only before the eighth month of pregnancy. However, the exact reason why the anterior presentation predominates remains unclear.

Turbidity seen from about mid-gestation in the amniotic fluid may indicate increased secretions from the buccal cavity and respiratory tract, leading to increased viscosity and improved lubrication (35). Adams-Brendemuehl and Pipers (36) identified echogenic particles in the allantoic fluid of all mares within 10 days of parturition, corroborating the findings of the current study.

The frontal view was the most suitable position for fetal sexing early in gestation in horses (17), cattle (17, 37), buffaloes (20), and camels (21). Depending on the location of the GT in the frontal view, the sex of the fetus in Arabian horses can be determined as early as the 56th day, with the optimal period falling between days 105 and 133. The best time was found to be between the 10th and 13th weeks of pregnancy in cattle (38), the 10th and 18th weeks in buffaloes (20), and the 11th week in camels (21). The sex could not be determined before the 56th day due to a lack of clarity or the presence of the GT just between the hind legs. The GT was sometimes unclear due to an inappropriate fetal position or the presence of numerous hyperechogenic structures in the examination area. We were also unable to determine the fetus’s gender after the 161st day because it was out of the scope of the ultrasound. By mid-gestation, the scrotum or the mammary glands could be observed between the two hind limbs. Renaudin et al. (18) found that the mammary glands and teats were visible in equine fetuses between 118 and 227 days of gestation. Curran and Ginther (17) were correct in their fetal gender diagnosis in 97% of males and 100% of females when they could locate the GT relative to the tail and umbilical cord, which is comparable to our findings.

While the findings provide valuable preliminary insights into fetal development patterns in Arabian mares, they should be interpreted with caution until validated in larger samples. Future studies involving larger cohorts across various ages and management systems are needed to confirm and expand upon the current findings.

## 5 Conclusion

This study provides preliminary reference ranges for transrectal ultrasonographic measurements to estimate gestational age in Arabian horses. The regression models could assist veterinarians in predicting parturition; however, the limited sample size calls for cautious interpretation and validation in larger populations.

## Data Availability

The original contributions presented in the study are included in the article/supplementary material, further inquiries can be directed to the corresponding author.
